# Effect of Drying Methods on Bioactive Compounds and Antioxidant Capacity in Grape Skin Residues from the New Hybrid Variety “BRS Magna”

**DOI:** 10.3390/molecules25163701

**Published:** 2020-08-14

**Authors:** Gabriela Viana da Silva, Bruna Aparecida Souza Machado, Walkia Polliana de Oliveira, Camilla Fernanda Godinho da Silva, Cedenir Pereira de Quadros, Janice Izabel Druzian, Ederlan de Souza Ferreira, Marcelo Andrés Umsza-Guez

**Affiliations:** 1 School of Pharmacy, Federal University of Bahia (UFBA), Salvador 40170-115, Bahia, Brazil; gvstecnologa@yahoo.com (G.V.d.S.); walkia2010@hotmail.com (W.P.d.O.); camillagodinho@gmail.com (C.F.G.d.S.); janicedruzian@hotmail.com (J.I.D.); 2 Technology College, National Service for Industrial Learning (SENAI/CIMATEC), Salvador 41650-010, Bahia, Brazil; brunam@fieb.org.br; 3 School of Pharmacy, Federal University of the São Francisco Valley (UNIVASF), Petrolina 56300-000, Pernambuco, Brazil; cedenir.quadros@univasf.edu.br; 4 Department of Biotechnology, Health Science Institute, Federal University of Bahia (UFBA), Salvador 40170-115, Bahia, Brazil

**Keywords:** *Vitis vinifera*, agro-food waste, polyphenols, conventional oven, freeze-drying

## Abstract

The effects of the drying process using the conventional oven and freeze-drying on the thermogravimetric profile, proximate composition, color parameters, individual bioactive compounds, and antioxidant activity in the grape residue (skin) were evaluated. Twenty individual phenolic compounds were identified, where a variation in concentration was observed for flavonols, stilbenes, phenolic acids, flavonoids, procyanidins, and particularly anthocyanins (malvidin-3,5-di-*O*-glucoside of 253.2–536.9 mg/kg) due to the drying process. Drying in a conventional oven caused a decrease of 23% of the total polyphenols. The skin of the BRS magna grape has a high concentration of total phenolic content of 489.5–148.3 mg.GAE/100 g, total anthocyanin content of 124.9–260.1 mg.CE/100 g, and total flavonoid content of 12.7–26.0 mg.QE/100 g. The results of free radical scavenging activity (1.26–4.91 μg/mL, as EC_50_) and ferric reducing antioxidant power (82.93–184.10 μmol/g of skin as equivalent to Fe_2_SO_4_) indicate high antioxidant activity, independently of the drying process applied. It was concluded that, if the application is directed to anthocyanin compounds, the use of lyophilization is recommended. On the other hand, if the interest is in bioactive compounds that exert antioxidant activity, conventional oven-drying can be used.

## 1. Introduction

Today, the challenge of the social, economic, and environmental impacts of food waste has become an urgent issue globally. The main causes of food waste or loss are the production, processing, retail, and consumption stages [1].

In this sense, the sustainable exploitation of waste will be a useful strategy with the aim of reducing environmental contamination and as an alternative to reducing the carbon footprint in the entire production process [2]. In addition, sustainable solid waste management is crucial for the prevention of infectious diseases, as risk factors for vector-borne diseases [3]. One of the current challenges is the processing of these residues for the elaboration of new products, used as ingredients [4], improvers of technological-functional properties [5], and for nutritional enrichment [6], which can reduce its negative impacts and obtain products with high added value [7]. In this context, the viticulture industry is included, as it generates a large amount of waste. Approximately, 20–30% of the weight of the processed grapes (juice, jams, wine, and raisins) remains as waste, consisting mainly of parts of stem, skin, and fruit seeds. Therefore, the wine industry produces millions of tons of waste per year, which represents a waste management issue, both ecologically and economically [8].

Grape skins are the main residue of viticulture, considered a great source of polyphenol compounds, such as flavonoids, anthocyanins, phenolics, and resveratrol. In fact, grape residue (skin) has a considerable amount of bioactive substances. This residue is attributed several protective and biological effects, such as antioxidant, antimicrobial, anti-tumor, anti-obesity, and anti-diabetes effects, in addition to helping to prevent neurodegenerative and cardiovascular diseases and having a prebiotic function in the intestinal microbiome [9,10,11].

The presence of these bioactive compounds in grape skins residues can add value due to the potential for more noble applications, whether in the food, pharmaceutical, or cosmetic industry [7]. Therefore, there is interest in applying grape by-products to fortify foods or as ingredients in products that contain high levels of bioactive compounds with these possible health benefits [12]. In addition to being attractive to health, the extracts have been widely used for the preparation of dehydrated natural dyes, mainly anthocyanins and applied to foods [13]. However, it is essential to know the chemical characteristics of the matrix (grape skin) and, above all, to understand the techniques for extracting these compounds, avoiding significant losses/damages to the compounds of interest [14].

Generally, dehydration techniques are commonly applied for the treatment of grape skin residues, taking into account the conditions of the process to preserve the compounds of interest (polyphenols). The most traditional method is dehydration in a conventional drying oven under different temperature and time conditions [15]. However, due to the long drying time often required, these methods can cause hydrolysis, oxidation, and degradation of the compounds of interest [16]. Some studies have evaluated the effects of different methods of thermal dehydration on color, phenolic, and antioxidant properties of fruit skins [5,17,18].

Some unconventional methods, such as dehydration by freeze-drying and spray-drying methods, have been proposed to maintain the total content of polyphenols and the antioxidant effect of fruits and their derivatives, due to the preservation of thermolabile compounds, and to promote the conservation of longer storage times [5]. However, there is little information about the effect of drying temperatures used in the profile of the phenolic compounds of interest present in grape skin residues, and correlations with other parameters (color, antioxidant capacity, etc.), especially in recently developed hybrid varieties.

The “BRS magna” grape is a new hybrid variety obtained from the genetic crossing of “BRS Rubea” and “IAC 1398-1321 (Traviú)” developed by the Brazilian Agricultural Research Corporation used mainly for juice production in the São Francisco Valley region (between Petrolina and Juazeiro, Brazil), but there is still not much information about this variety, including the by-product (bark). It is a possible raw material for research on its use in the preparation of new foods or as a fortifying ingredient (bioactive compounds). Therefore, the aim of this study was to compare the effects of dehydration using the conventional oven-drying and freeze-drying process on the polyphenols content and antioxidant capacity in “BRS magna” grape skin residues. Quantitative and qualitative changes in individual phenolic compounds in fresh and dehydrated samples were determined using HPLC-DAD-FD. In addition, alterations in color parameters; content of total phenolics (CTP), total anthocyanins (CTA), and total flavonoids (CTF); antioxidant activity; and the correlation among these were also evaluated.

## 2. Results and Discussion

Currently, an incipient part of the residues derived from industrial grape processing is used to produce different products, from the process of re-fermentation of beverages and distillation, animal feed, and fertilizers [7]. However, the largest amount is discarded and incinerated, making it an important economic issue, in addition to a significant environmental problem [3]. On the other hand, these agri-food residues (fruits, vegetables, etc.) present an opportunity for commercial exploitation [17], in the form of new products in the food industry (natural dye, as a food/nutritional quality enhancer) [9,11,13,19,20]; in the pharmaceutical industry (functional supplements) [21,22,23]; in the cosmetic industry (products with antioxidant and photoprotective effects and inhibition of dermal proteases) [24].

In fact, some studies have shown the use of grape skins (fresh or dehydrated) as a supplementary source of phenolic compounds [4] in food products (e.g., bakery products such as cereal bars, muffins, and cookies; dairy products such as cheese and yogurt; and seafood, purees, and infusions) [25], while grape skin extracts can be used in drinks [5]. However, the exploitation of grape residues is scarce mainly due to its high moisture content, as it causes deterioration and degradation of valuable compounds [12].

### 2.1. Proximate Composition

In this study, the effects of dehydration were compared using conventional oven-drying and the lyophilization process on the content of polyphenols and the antioxidant capacity in grape skin residues of the “BRS magna” variety. The proximate composition of the BRS magna bark residues is shown in Table 1. There were no statistically significant differences (*p* > 0.05) between the skins in the parameters of ash, crude proteins, total lipids, fibers, and carbohydrates, which shows that the drying procedures did not cause degradation or change in the composition of the residue, as they preserved nutritional attributes.

The residue has a high amount of carbohydrates (about 83.5%), of which a considerable part of fibers (about 38%), proteins (about 7%), and ash (about 6.5%). Because of this, some studies have developed low-calorie foods, with a higher content of fiber, ash, and protein, using fruit skin residues to enrich these products [4,6,26]. Recently, Falcão et al. [6] elaborated an umbu bark jam (*Spondias tuberosa*) and Amorim et al. [4] developed a functional food ingredient enriched with polyphenols (extract based on grape skin variety *Syrah*) that showed a significant increase in nutritional properties, i.e., protein, ash, and concentrations of total phenolic compounds.

### 2.2. Thermogravimetric Analysis

**Figure 1:**
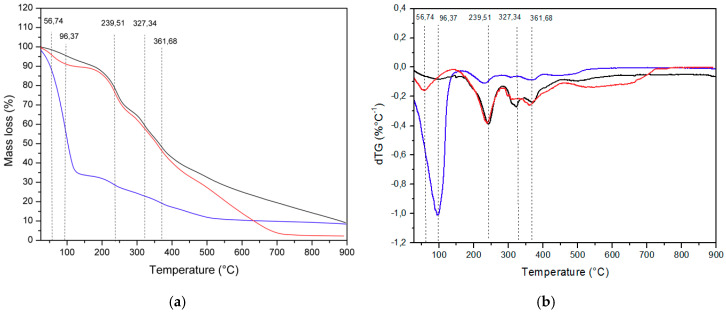
Thermogravimetric curve (TG) analysis (**a**) and for derivative weight dTG (**b**) of dehydrated samples of BRS magna grape skin residue using conventional oven-drying and freeze-drying methods. The profiles shown as blue, red, and black lines represent the fresh skin, conventional oven-drying, and freeze-drying processes, respectively.

Figure 1 traces the profile of TG and DTG curves during the combustion process of “BRS magna” skin residues. There were differences in the thermogravimetric parameters between the fresh skin and the materials obtained by drying (*p* < 0.05), but between the last two the behavior was similar. One explanation for the difference observed is that the fresh skin residue has the highest moisture content (76.1 ± 0.7%) when compared to other residues, which were subjected to conventional drying at 65 °C (4.6 ± 0.1%) and dehydration by lyophilization (9.0 ± 0.1%).

As can be seen in Figure 1a, the first thermal mass loss event (TG) occurred at a temperature of 96 °C (onset), with a weight loss of 66.9 ± 3.7% and 23.2 ± 1.5%; then at 239 °C (onset), with a 9.5 ± 0.2% reduction in mass and 27.1 ± 1.9%; close to 327 °C (onset), with a decrease of 4.3 ± 0.3% and 12 ± 0.7%; and finally at 362 °C (onset), with a reduction of 11.9 ± 1.1% and 37.9 ± 2.6%, respectively, for fresh and dried samples (*p* < 0.05). The samples submitted to conventional drying and dehydration by lyophilization showed a smooth curve, indicating that the materials underwent decomposition during the formation of gaseous products. These results are close to those found by Gowman et al. [27] for the TG behaviors of grape and apple residues generated during the production of juice and wine, which also observed smooth curves, with the presence of peaks due to the evaporation of water and its other components.

In Figure 1b, the DTG curves are shown, where it was possible to observe that there are four well-defined mass losses, which allow the identification of the combustion stages [28]. The first derivative is attributed to the drying stage, occurring in the moisture content of the skin waste evaporation, characterized by a peak around 102 °C in the DTG curves (see Figure 1b). In the second and third events at the temperature range of 190–320 °C, the devolatilization phase occurred, which is characterized by a peak of around 246 °C, in the DTG. In this stage, the peak is due to the oxidation of hydrogen present in organic matter, as well as the release of volatile compounds from this decomposition. In this sense, the peaks observed at the beginning of 214 °C and 311 °C in the DTG are attributed to the degradation temperatures of hemicellulose and cellulose, respectively [27]. The fourth thermal event at 372 °C in the DTG marked the beginning of the coal oxidation stage [28], but a discreet and poorly defined peak was observed compared to the main ones. Even at this stage, the peak is due to the degradation temperature of lignin. These results are close to those reported for the DTG for grape and apple residues that showed peaks due to the degradation of hemicellulose at 267 and 260 °C, cellulose at 340 and 335 °C, and lignin at 378 and 383 °C, respectively [27]. The dry skin residue of the BRS magna grape, regardless of the dehydration process carried out (conventional oven-drying or freeze-drying), proved to be thermally stable for most applications in industries, which are dependent on temperature.

### 2.3. Color Parameters

Table 2 shows the changes in color parameters due to the effect of oven-drying and freeze-drying. All samples showed characteristic red grape color in the values of L*, C*, and H°. However, there were statistically significant differences between treatments, where it is observed that the L* and C* indexes varied from 32.8 to 39.3 and 7.0 to 10.7, which represented increases of 6.4% and 19.8% (*p* < 0.05) and 8.6% and 52.9% (*p* < 0.05) in oven-dried and freeze-dried materials, respectively, compared to fresh skin.

These results are close to those found by Natividade et al. [29] for parameter L* with values from 38.3 to 38.9 for the residues of Isabel grapes dried by oven and freeze-drying methods, respectively. In addition, Pedroza et al. [5] showed that drying in an oven at 60 °C promoted changes in the shade of red (from 0% to 15%) and color intensity (from 0% to 100%) of the skin residues Bobal, Garnacha Tintorera, and Cabernet Sauvignon grapes. In another study, Ruttarattanamongkol et al. [17] observed that drying by hot air at 70 °C in orange-fleshed sweet potato and purple-fleshed sweet potato increased L* values by about 9–74% compared to those in natura. There were no differences in the H° parameters between oven-drying and freeze-drying.

Color loss is a natural process that occurs with all types of fruits and vegetables due to chemical and biochemical transformations related to the content of polyphenols and the presence of oxygen [13]. However, these changes are particularly important for food, because color is an important quality parameter that can influence consumer preferences [6]. Despite this, many agro-residues can be used as a source of dehydrated pigments and also have other effects on food products, e.g., antioxidant and antimicrobial activity [20,23].

Previous studies on dehydration and characterization of grape skins residues showed favorable parameters for the release of color compounds, polyphenols, and aromas [4,5,12,16]. Thus, it is possible to observe that both the dehydration process by conventional oven-drying and freezing improved the color attributes of the BRS magna grape skin residues. Pedroza et al. [12] evaluated the addition of different dehydrated grape skins in young and aged reds to compensate for the loss of color before bottling. The results show that, after adding the skins, the color intensity of the wines increased by an average of 11% and a maximum of 31%, with a predominance of the red component. In addition, the average total increase in polyphenols was 10%, with a maximum value of 20%. In this study, mixtures of dehydrated grape skins were useful to improve the color and polyphenols profile of red wines, considering them as a useful tool to correct color loss before bottling.

### 2.4. Identification and Quantification of Phenolic Compounds

Table 3 shows the effect of conventional kiln drying and freeze-drying on individual phenolic compounds from the BRS magna grape skin residues. It is possible to observe from the total of all quantified compounds that conventional oven-drying (*p* < 0.05) caused a 23% decrease in the total polyphenols. However, this effect was not seen in the lyophilization process.

Twenty-seven phenolic compounds were identified in the samples. There were statistically significant differences between treatments, with variation between anthocyanins, flavonols, stilbene, phenolic acids, flavanols, and procyanidins.

Anthocyanins represented the main group of compounds in the skins, but different proportionalities were observed: 58.6%, 36.3%, and 82.2% (*p* < 0.05) for fresh skin, oven-drying, and freeze-drying, respectively, as reported in other studies [30]. This can be explained due to an effect observed in conventional oven-drying, which provided a 52% reduction in the compounds, while there was a 41% increase in lyophilization compared to the fresh skin on a dry basis. Malvidin-3,5-di-*O*-glucoside represented the main compound among anthocyanins at 68%, 67%, and 76%, and among all compounds found in the skin at 40%, 25%, and 63% for fresh skin (536.9 mg/kg), oven-drying (253.2 mg/kg), and lyophilization (848.3 mg/kg), respectively. Other anthocyanins found in significant quantity were delphinidin-3-*O*-glucoside (37.1–115.9 mg/kg), petunidin-3-*O*-glucoside (25.5–45.3 mg/kg) cyanidin-3-*O*-glucoside (13.1–59.3 mg/kg), and malvidin-3-*O*-glucoside (13.5–35.7 mg/kg).

Flavanols were the second set of major compounds in phenol group whose concentration was 13.9%, 13.8%, and 6.5% for fresh skin (185.5 mg/kg), in oven-drying process (142.3 mg/kg), and freeze-drying (87.4 mg/kg), respectively, among which the compounds epicatechin gallate (35.8–68.8 mg/kg) and epigallatocatechin gallate (22.7–68.8 mg/kg) were the main representatives. In addition, there was a statistically significant decrease (12.8% and 19%) in the amount of flavonoids between fresh skin and materials obtained by conventional drying and freeze-drying (*p* < 0.05), but not between the drying processes (*p* > 0.05).

Quercetin-3-β-d-glucoside was the main component of flavonols, varying its concentration from 17 to 138 mg/kg, which represented 64.9%, 74.3%, and 43.1% of the total fresh skin compounds, conventional drying, and freeze-drying, respectively. With the exception of quercetin-3-β-d-glucoside and myricetin, all other compounds in this group had a reduction caused by the drying process (*p* < 0.05), e.g., kaempferol-3-*O*-glucoside of 39.7% and 68.7%, rutin of 28.4% and 69.4%, isorhamnetin-3-*O*-glucoside of 36.3% and 51.6%, and *trans*-resveratrol of 33.3% and 83.3% due to conventional drying and freeze-drying, respectively, compared to fresh peel on a dry basis.

The main phenolic acids found were *p*-coumaric (15.5–33.1 mg/kg), caftaric (24.6–86.0 mg/kg), and gallic acids (15.3–30.5 mg/kg). However, this group represented only 10.2%, 16.7%, and 5.2% of the total polyphenols that make up the bark residue of the BRS magna grape. The negative drying effects can be seen due to the lower concentration of gallic, ferulic acid, chlorogenic, and *p*-coumaric acids in 25.6% and 49.8% (*p* < 0.05), 42.6% and 67.8% (*p* < 0.05), 0.5% (*p* > 0.05) and 53.2% (*p* < 0.05). A similar effect was observed in procyanidin A2, which decreased 24.9% and 72% in conventional drying and freeze-drying, respectively, compared to fresh peel on a dry basis. On the other hand, conventional drying caused an increase in the amount of procyanidin B1 and procyanidin B2 compounds of 71.9% and 288.5%.

In recent years, several studies have been carried out to evaluate the effect of different drying methods (thermo-degradation/thermo-protection) on grape phenolic compounds, grape marc [5,16,31]. These studies, according to the results presented in this work, affirm that the drying temperature below 70 °C and the lyophilization process preserve a good part of the bioactive compounds (phenolics).

### 2.5. Total Phenolic, Anthocyanin, and Flavonoid Contents and Antioxidant Activity

Table 4 shows the effect of conventional and freeze-drying on total phenolic compounds, total flavonoids, total anthocyanins, and antioxidant activity of BRS magna grape skin residues. The skin residues of fresh BRS magna grapes showed a high concentration of total phenolic content (TPC, 489.5 ± 1.8 mg/100 g of material, as GAE), total anthocyanin content (TAC, 124.9 ± 2.7 mg/100 g of material, as CE), and the total flavonoid content (TFC, 26.0 ± 0.6 mg/100 g of material, as QE). However, TPC and TFC had a reduction in comparison with the residues of fresh peel (control) of 69.3% and 69.7% and 31.2% and 51.2% (*p* < 0.05), but an increase in TAC of 20.7% and 108.3% (*p* < 0.05) in conventional drying and lyophilization, respectively. The variation in the levels of polyphenols and antioxidant activity according to the drying conditions applied has been observed in previous studies [5,15,18,32]. The conditions of thermal processing apparently have a significant effect on these parameters. Heat treatment can cause damage to the cellular structures of grape pulp tissues and result in easier extraction of antioxidant constituents [17]. It is interesting to note that the contents of some compounds after the drying processes were significantly (*p* < 0.05) higher when compared to fresh skin and freeze-drying sample, i.e., quercetin-3-β-d-glucoside, caffeic acid, and procyanidins, as shown in Table 3 and supported by the results of antioxidant activity (Table 4).

The amount of TPC observed in the present study was greater than in some grape varieties. Lago-Vanzela et al. [33] for the Bordô grape (*Vitis labrusca)* reported TPC values 113.0 mg.GAE/100 g of fresh grape, being 103.4 mg.GAE/100 g (93.7%) in the skin and 9.6 mg.GAE/100 g (6.3%) in the pulp. Lima et al. [34] for juices produced using the varieties Isabel Precoce, BRS Cora, BRS Violeta, BRS Magna, Blend Isabel Precoce (80%) and BRS Violeta (20%), and Blend Isabel Precoce (80%) and BRS Cora (20%) found TPC values around 77.9–271.2 mg.GAE/100 mL. In addition, Pintać et al. [35] reported an amount of TPC in grape marc extracts (80% EtOH) of 426, 193, 237, and 287 mg.GAE/100 g for Cabernet Sauvignon, Merlot, Italian Riesling Agner, and Italian Riesling Bajilo, respectively. However, Tavares et al. [15] recently reported higher TPC values of 815, 834, and 898 mg.GAE/g for BRS Violet bagasse even after drying on a foam mat at 60, 70, and 80 °C, respectively. Rockenbach et al. [36] showed that the grape skins of the varieties Isabel, Cabernet Sauvignon, and Primitivo showed considerable high concentrations of phenolics, around 1065–1839 mg.CE/100 g, while the values of Pinot Noir, Sangiovese, and Negro Amaro ranged from 660 to 750 mg.CE/100 g, which are values close to those found in this work for the fresh skin residues of the BRS magna grape (Table 4).

Anthocyanins are the main group of polyphenolic compounds in the BRS magna grape skins, as shown in Table 3 and Table 4. Evidently, this characteristic is due its origin deriving from the crossing of the varieties “BRS Rúbea” and “IAC 1398-21 (Traviú)”, both with characteristics of violet color [34]. Therefore, the skin of this new hybrid grape variety can be considered an excellent source for exploring this component [7,13]. The TAC observed in the skin was close to the values reported by Lago-Vanzela et al. [33] for the Bordô grape skin (*Vitis labrusca)* of 135.9 mg/100 g, as malvidin-3,5-diglucoside equivalents. However, it was superior to the results presented by Lima et al. [34] for grape juices produced from six new Brazilian varieties planted in the Northeast region of Brazil, including BRS magna, which presented concentrations in the range of 0.6–17 mg/100 mL, including cyanidin-3,5-glucoside. Anthocyanins represent the main group of polyphenols, as reported in previous studies in other peels of violaceous grapes [34,36].

In this study, the TFC values were higher than those observed by Lima et al. [34] of 7.2–11.1 mg/100 mL for juices produced by different varieties. However, it was similar to those reported by Pintać et al. [35] for Merlot bagasse extracts of 24 mg.QE/100 g, and less than that found (65–74 mg.QE/100 g) in Cabernet Sauvignon, Italian Riesling Agner, and Italian Riesling Bajilo. In addition, Isabel, Sangiovese, Cabernet Sauvignon, and Primitivo grapes presented TFC concentrations that ranged from 156 to 252 mg/100 g, as equivalent to catechin [36].

The results of DPPH and FRAP indicate that the BRS magna grape residue extract showed high antioxidant activity in the fresh skin, as well as in the materials obtained by drying in the oven and in the lyophilization process (Table 4). The antioxidant activity (DPPH and FRAP) was higher in the skins obtained by conventional drying, as seen in Table 4. The phenolic acids (gallic acid, caftaric acid, caffeic acid, ferulic acid, chlorogenic acid, and p-coumaric acid) and flavanols (epicatechin, epicatechin gallate, epigalatocatechin gallate, and catechin) are compounds related to antioxidant activity (Table 3). These have higher values than those obtained by freeze-drying. In addition, as shown in Table 4, the value of total flavonoids was higher in samples obtained from oven-drying.

These assays have been widely used to assess the antioxidant capacity of extracts from different foods and products [4,15,17,18,31]. The reduction of the DPPH free radical is determined by decreasing its absorption when the color of the DPPH test solution changes from purple to light yellow. Therefore, the antioxidant potential of grape residue extract corresponds to the degree of discoloration [18], while the reduction in the ability to transfer electrons to a Fe^3+^/tripyridyl triazine reagent measures the antioxidant power by the FRAP method [15].

The DPPH of the fresh skin residues of the BRS magna grape was 1.7 ± 0.2 μg/mL, as EC_50_, and the FRAP was 184.1 μmol/g of bark residues, as equivalent to Fe_2_SO_4_. A decrease in antioxidant power was observed by DPPH (3.8 ± 0.1 μg/mL and 4.9 ± 0.1 μg/mL, as EC_50)_ and FRAP (163.5 ± 10.9 μmol/g and 82.9 ± 9.3 μmol/g of skin as equivalent to Fe_2_SO_4_) due to conventional drying and lyophilization, respectively. The decline in antioxidant activity is partially related to the reduction in the concentration of polyphenols, as well as to the phenomena of oxidative degradation and polymerization–condensation of some compounds [5,16]. In addition, the results observed in the present study are in agreement with other studies reporting that the phenolic content and antioxidant activity were affected by cooking processes, such as steam, boiling, and drying. Variations in total phenolic content and antioxidant activity according to drying conditions have been reported previously [17,18,32].

Recently, Ramón-Gonçalves et al. [32] observed that the temperature influenced the conditions of experimental extraction to obtain the maximum response of polyphenols from used coffee beans. The extraction was carried out with different hydroalcoholic solutions (EtOH:H_2_O, 50:50, 40:60, and 30:70, *v/v*), modification times (15, 23, or 30 min) and temperatures (80, 100, and 120 °C). The extraction of the three conditions using the lowest temperature of 80 °C exhibited the highest content of polyphenols and antioxidant activity, where the best results were obtained at the temperature of 80 °C for 30 min with a mixture of EtOH:H_2_O, 50:50 (*v/v*). In addition, some compounds have been shown to have a greater influence on temperature during extraction (e.g., rutin). The extracts obtained under these conditions are characterized by high values of the total flavonoid content, in the range of 110–560 mg.QE/100 g dw and total polyphenol content of 140–450 mg.GAE/100 g dw. Planinić et al. [31] showed that drying at higher temperatures and a prolonged process have a greater impact on antioxidant activity, when compared to drying treatment at milder temperatures and shorter periods. Grape marc extracts that were dried for 180 min at 80 °C showed 20% less antioxidant activity, while the grape marc sample dried for 90 min at 60 °C had 11.43% lower neutralization capacity (DPPH), compared to fresh peel extract. In the present study, the antioxidant activity of the grape skin residue was influenced by thermal reactions [5], possibly due to the polyphenols of low molecular weight that were generated from the enzymatic hydrolysis of polymeric polyphenols from the cellulose matrix [37].

### 2.6. Correlation between Color Parameters and Antioxidant Activity with Phenolic Compounds

**Figure 2:**
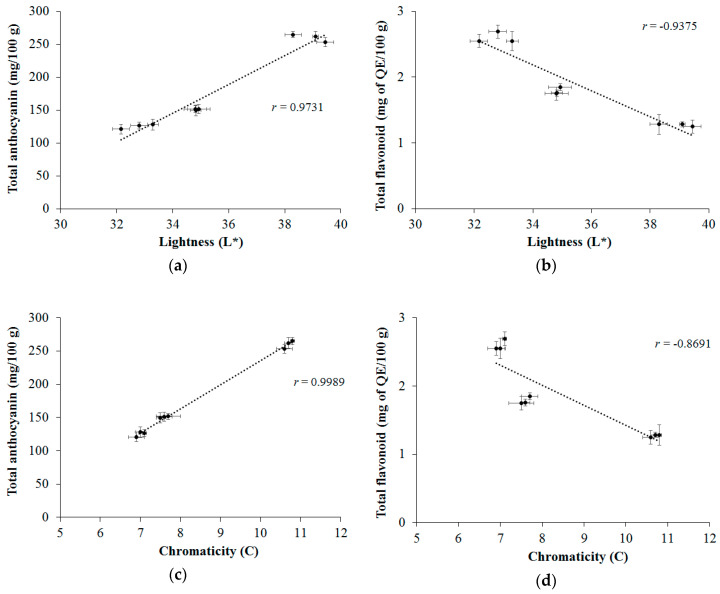
Correlations between: luminosity and TAC (**a**), luminosity and TFC (**b**), chromaticity and TAC (**c**) and chromaticity and TFC (**d**). The values of mean ± standard deviation (SD) correspond to averages from three samples.

Figure 2 shows the *r* values for Pearson’s correlation between the contents of bioactive compounds and the results of the color parameters, depending on the treatment. There is a high correlation (above 0.9) between the brightness parameter for TAC (Figure 2a) and TFC (Figure 2b), but the correlation was positive for TAC (*r* = 0.9731) and negative for TFC (*r* = −0.9375), as an effect of heat treatment. Close results were also observed between the chromaticity parameters with TAC (Figure 2c) and TFC (Figure 2d). The degree of correlation between these parameters has also been reported by previous studies [4,33].

**Figure 3:**
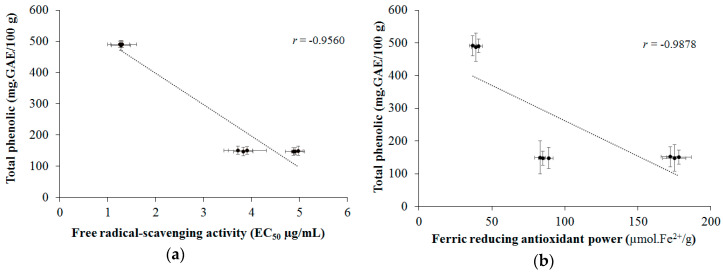
Correlation: between TPC and the DPPH radical scavenging assay (**a**); and between TPC and FRAP assay (**b**). The values of mean ± standard deviation (SD) correspond to averages from three samples.

Figure 3 shows the *r* values for the correlation between the total phenolic content and the antioxidant activity. A high correlation (*r* = −0.9560) was observed between the total phenolic content and the free radical scavenging activity (Figure 3a) and the reducing ferric antioxidant power (Figure 3b). Important correlations between phenolic compounds and the antioxidant capacity of grape residue extracts have been observed in other studies [37,38] as well as in the present study.

Martins et al. [37] reported a correlation between polyphenolic compounds (quercetin, *trans*-resveratrol, gallic acid, caffeic acid, caffeic acid, catechin, and procyanidin B2) and antioxidant activity in white grape peel in the ranges of 0.414–0.778 for DPPH and 0.899 to insignificant ranges for FRAP results. Although the increase in DPPH values was correlated with all the phenolic compounds analyzed, FRAP values were correlated to an even stronger degree. Given that *r* values ≥ 0.6 indicate a significant correlation, only two polyphenolics, gallic acid and resveratrol, were correlated to all three tests for antioxidant activity. Sir Elkhatim et al. [38] showed a high correlation between antioxidant activity (DPPH) and the level of total phenolic (0.9303), as well as lower correlations with the values of total flavonoids (*r* = 0.111) and vitamin C (*r* = 0.2021), in residues of citrus fruits (grapefruit, lemon, and orange). However, different results were found by Xu et al. [39] who observed in grape marc a positive correlation of ABTS elimination capacity with the values of TPC and TFC (*r* = 0.798 and 0.977), but not with DPPH (*r* = 0.018 and −0.409), respectively. Only TAC showed a positive correlation with DPPH (*r* = 0.991). It has been suggested that different phenolic compounds are responsible for tempering different free radicals: flavonoids, tannins, and condensed tannins contribute to the antioxidant capacity of ABTS, while anthocyanins contribute to DPPH.

## 3. Materials and Methods

### 3.1. Materials

Folin–Ciocalteau reagent, 2,2-diphenyl-1-picrylhydrazyl (DPPH), 2,4,6-Tris(2-pyridyl)-1,3,5-triazine (TPTZ), cyanidin-3,5-di-*O*-glucoside, malvidin-3,5-di-*O*-glucoside, pelargonidin-3-*O*-glucoside, delfinidin-3-*O*-glucoside, cyanidin-3-*O*-glucoside, malvidin-3-*O*-glucoside, peonidina-3-*O*-glucoside, petunidin-3-*O*-glucoside, kaempferol-3-*O*-glucoside, rutin, isorhamnetin-3-*O*-glucoside, myricetin, *trans*-resveratrol, quercetin-3-β-d-glucoside, gallic acid, caftaric acid, caffeic acid, ferulic acid, chlorogenic acid, *p*-coumaric acid, (–)-epicatechin, (–)-epicatechin gallate, (–)-epigalatocatechin gallate, (+)-catechin, procyanidin A2, procyanidin B1, and procyanidin B2 compounds were purchased from Sigma-Aldrich^®^ (St. Louis, MO, USA). The solvents (methanol, acetonitrile, and ortho-phosphoric acid) used in the extraction and HPLC procedures were analytical/HPLC grade and purchased from Merck^®^ (São Paulo, Brazil).

### 3.2. BRS Magna Grape Skin Residue and the Dehydration Process

The “BRS Magna” grape skins obtained during the 2018 harvest were kindly supplied by a juice company in the São Francisco Valley (9°23′34″ S, 40°30′28″ W, Brazil) and transported in a plastic container at 4 °C to the Food Science Laboratory of the Faculty of Technology (National Industrial Training Service—SENAI/CIMATEC, Salvador, Brazil). The husks were manually separated from the other parts (seeds and stems). The material was packed and stored in freezing conditions (−70 °C) until the dehydration process was applied. The residues of grape skins “BRS Magna” were dried conventionally, being placed in trays, 2.5 cm thick, in an oven-drying process with air circulation (Q314M, Quimis^®^, Diadema, Brazil) at 65 °C to constant weight. In dehydration by lyophilization, the sample was left in the Liobras^®^ equipment (model L108, LIOTOP, São Carlos, Brazil) at a vacuum pressure of 4 × 10^−2^ mBa at a temperature of −57 °C for 24 h. Frozen samples of fresh skin (in natura) were used as a control treatment in order to evaluate the effect of dehydration methods on composition, color, phenolic compounds, and antioxidant capacity.

### 3.3. Proximate Composition

The materials were ground (with a 495 mm sieve) to obtain a uniform particle size. These analyses were performed according to the established methods [40]. Briefly, the moisture content was determined by oven-drying (Q314M, Quimis^®^, Diadema, Brazil) at 105 °C until constant weight. The crude protein content was calculated by the total nitrogen content (*N* × 5.3), using nitrogen distiller equipment (TE036/1, Tecnal^®^, Piracicaba, Brazil). The ash content was obtained in a muffle at 550 °C (SP2707-21, Quimis^®^, Diadema, Brazil). The total lipid content was determined by gravimetric analysis after extraction in a Soxhlet device (TE188/6, Tecnal^®^, Piracicaba, Brazil), using petroleum ether for 6 h. The amount of crude fiber was determined through sequential acid (1.25% sulfuric acid) and alkaline (1.25% sodium hydroxide) treatments. The carbohydrate content was estimated by calculating the percentage remaining after measuring all other components: % carbohydrates = 100 (moisture + protein + lipid + ash). The results are presented in g/100 g of material on a dry weight (dw).

### 3.4. Color Parameters

Color was evaluated using the parameters CIE Lab for luminosity (L*), hue by hue angle (H°), and color intensity by chromaticity (C), using a 2 mm bucket length in a CR-400 colorimeter (Konica Minolta^®^, Tokyo, Japan), following the manufacturer’s recommendations.

### 3.5. Individual Phenolic Compounds by HPLC-DAD-FD

Individual phenolic compounds were analyzed by high performance liquid chromatography (Waters 269, Aliance System^®^, Water Corporation, Milford, USA) equipped with a diode array detector (DAD) and a fluorescence detector (FD). The samples were previously filtered through a 0.45-μm nylon membrane (Phenomenex^®^, Torrance, CA, USA) and injected in triplicate (10 μL). The conditions were: pre-Gemini-NX C18 column (4.0 mm × 3.0 mm, Phenomenex^®^, Torrance, CA, USA), Gemini-NX C18 column (150 mm × 4.60 mm × 3 μm, Phenomenex^®^, Torrance, CA, USA), temperature from oven at 40 °C, and flow rate of 0.5 mL/min of mobile phases, consisting of (A) 0.85% phosphoric acid solution and (B) acetonitrile. The elution gradient was used as follows: 0 min, 100% A; 10 min, 93% A; 20 min, 90% A; 30 min, 88% A; 40 min, 77% A; 45 min, 65.0% A; and 100% B at 55 min. DAD was used at wavelengths 280 nm (gallic acid, (–)-epicatechin gallate and (–)-gallate epigallatocatechin), 320 nm (*trans*-resveratrol, caffeine, caffeine, caffeic, ferulic, chlorogenic, and *ρ*-coumaric acids), 360 nm (kaempferol-3-*O*-glucoside, myricetin, isorhamnetin-3-*O*-glucoside, rutin, and quercetin-3-β-d-glucoside), 520 nm (cyanidin-3,5-di-*O*-glucoside, malvidin-3,5-di-*O*-glucoside, pelargonidin-3-*O*-glucoside, delphinidin-3-*O*-glucoside, cyanidin-3-*O*-glucoside, malvidin-3-*O*-glucoside, peonidin-3-*O*-glucoside, and pethunidine-3-*O*-glucoside), and fluorescence with 280 nm excitation and 320 nm emission ((−)-epicatechin, (+)-catechin, procyanidin A2, procyanidin B1, and procyanidin B2) were used to identify and quantify the compounds. The identification and quantification of phenolic compounds was carried out as a method previously established [29]. The calibration curves were prepared using standards for 27 phenolic compounds (see Figure S1). The linearity of the method consisted of different concentration ranges on the standard calibration curve (0.625–15.00 μg/mL). The regression coefficients equations (R^2^) ranged 0.9838–0.9999. The theoretical limits of detection varied from 0.001 to 0.190 μg/mL, while the theoretical limits of quantification (LOQ) varied from 0.003 to 0.370 μg/mL. The average recovery value ranged 98.27–102.01% (anthocyanins), 86.18–106.50% (flavonols), 83.97–100.93% (phenolic acids), and 86.86–97.10% (tannins). The accuracy of the RSD method ranged 0.73–2.87% for non-enriched samples and 0.71–9.24% for enriched samples. For RSD_R_, they were 1.99–6.46% for non-enriched samples and 1.34–9.26% for enriched samples. The results are presented in mg/kg, on a dw.

### 3.6. Content of Phenolics, Anthocyanins and Flavonoids

The total phenolic content (TPC) was determined spectrophotometrically (Beckman Spectrophotometer^®^ Coulter DU-70 UV/VIS, California, USA), according to the Folin–Ciocalteau method [41] through the absorbance measured at 750 nm. A calibration curve was constructed using standard gallic acid (25–200 μg/mL) to obtain the equation: y = 0.0073x − 0.0591 (R^2^ = 0.9999). The results are expressed in mg of gallic acid equivalent (GAE) per 100 g of samples, on a dw.

The total anthocyanin content (TAC) was determined according to the established method [42], using the absorbance measured at 535 nm (Beckman spectrophotometer^®^ Coulter DU-70 UV/VIS, Los Angeles, CA, USA). The total anthocyanin content was obtained using the equation: Total anthocyanin content mg/L = (*A* × *FD*)/(*ε* × *b)*, where *A* is the absorbance (535 nm), *ε* denotes the coefficient of absorbance cyanide-3-glucoside (26900, MW 449.2), *b* corresponds to the bucket thickness (1 cm), and *FD* refers to the extract dilution factor. The results are expressed in mg of cyanidin equivalent (CE) per 100 g of sample, on a dw.

The total flavonoid content (TFC) was determined by the aluminum chloride colorimetric method [43], using the absorbance measured at 415 nm (Beckman spectrophotometer^®^ Coulter DU-70 UV/VIS, Los Angeles, CA, USA). A calibration curve (5–35 μg/mL) was constructed from the quercetin standard from Sigma-Aldrich^®^ (CAS 6151-25-3, St. Louis, MO, USA) to obtain the equation: y = 0.0287x − 0.0076 (R^2^ = 0.9986). The results are expressed in mg of quercetin equivalent (QE) per 100 g of sample, on a dw.

### 3.7. Antioxidant Activity

The free radical scavenging activity (DPPH) was evaluated according to the established method [44], where the decrease in absorbance at 515 nm of DPPH 100 mM dissolved in 80% methanol is measured in 30 min after adding the sample. The antioxidant activity is expressed in EC_50_ μg per g of samples, on a dry weight. The ferric reducing antioxidant power (FRAP) method was evaluated in the direct measurement of antioxidant capacity (reducing) by reducing the Fe^3+^/tripyridyl triazine (TPTZ) complex to Fe^2+^ under acidic pH (3.6) [45]. The reduction capacity of the samples was determined using the absorbance at 620 nm (Beckman^®^ Coulter DU-70 UV/VIS, Los Angeles, CA, USA). The results are expressed as μmol.Fe^2+^ per g of sample, on a dw.

### 3.8. Statistical Analysis

The averages of the results were evaluated through the one-way analysis of variance (ANOVA). For multiple comparison, the Turkey test (SigmaStat, v. 3.5, Systat^®^ software, Chicago, IL, USA) was used. The level of significance was *p* ≤ 0.05. All results are presented as mean ± standard deviation for at least three independent analyses. Pearson’s correlation coefficient (r) was used to evaluate the covariance relationships between the content of bioactive compounds and the antioxidant properties of the skin residues of the BRS magna grape.

## 4. Conclusions

In this study, it was observed that the residue (skin) of the BRS magna grape variety has a high concentration of bioactive compounds and antioxidant activity. It was also found that conventional drying in an oven at 65 °C and freeze drying for 24 h caused significant changes in color parameters, composition of bioactive compounds, and antioxidant capacity at different intensities, but not in the proximate composition. In addition, it is worth mentioning that there were also positive changes, mainly the increase in antioxidant capacity. It was observed that the best drying method to be applied will depend on the purpose of its use. From the results obtained, while using conventional drying, the highest values obtained were among individual phenolic acids, which are mainly responsible for antioxidant activity (DPPH and FRAP), and among phenolic and total flavonoids. Freeze-drying was more effective in preserving anthocyanins, compounds that are known to be thermolabile.

## Figures and Tables

**Table 1:** Proximate composition of dehydrated BRS magna grape skin residue using conventional oven-drying and freeze-drying methods.

Parameters (% Dry Weight)	Method Applied ^‡^			
	Fresh Peel	Oven-Drying	Freeze-Drying	*p*-Value
Protein	7.0 ± 0.2	7.0 ± 0.8	7.2 ± 1.2	0.098
Ash	6.6 ± 0.2	6.4 ± 0.5	5.1 ± 0.6	0.058
Total lipids	2.9 ± 0.4	2.9 ± 0.1	3.3 ± 0.2	0.054
Total Carbohydrate ^†^	83.5 ± 0.8	83.8 ± 0.6	84.5 ± 0.9	0.063
Crude fiber	35.4 ± 2.0	38.0 ± 3.6	41.6 ± 1.8	0.082

^‡^ The values of mean ± standard deviation (SD) correspond to averages from three samples. ^†^ Defined by the difference between 100 and the sum of the percentages of other components, as shown in the Section 3.

**Table 2:** Effect of the drying process using conventional oven-drying and the freeze-drying considering lightness (L*), hue angle (H°), and chromaticity (C*) parameters of the BRS magna grape skin residue.

Parameters (% Dry Weight)	Method Applied ^‡^			
	Fresh Peel	Oven-Drying	Freeze-Drying	*p*-Value
L*	32.8 ± 0.6 ^c^	34.9 ± 0.1 ^b^	39.3 ± 0.2 ^a^	<0.001
C*	7.0 ± 0.5 ^c^	7.6 ± 0.1 ^b^	10.7 ± 0.1 ^a^	<0.001
**H°**	0.4 ± 0.1	0.5 ± 0.1	0.4 ± 0.1	0.420

^‡^ The values of mean ± standard deviation (SD) correspond to averages from three samples. Different letters on the same line indicate significant differences between the values (*p* < 0.05).

**Table 3:** Effect of the drying method using the conventional oven-drying and freeze-drying processes in 27 individual phenolic compounds of the BRS magna grape skin residue.

Phenolic Compounds (mg/kg)	Method Applied ^‡^			
	Fresh Peel	Oven-Drying	Freeze-Drying	*p*-Value
*Anthocyanins*				
Cyanidin-3,5-di-*O*-glucoside	27.9 ± 4.1 ^a^	11.8 ± 0.1 ^b^	34.0 ± 3.7 ^a^	<0.001
Malvidin-3,5-di-*O*-glucoside	536.9 ± 11.3 ^b^	253.2 ± 5.5 ^c^	848.3 ± 6.7 ^a^	<0.001
Pelargonidin-3-*O*-glucoside	23.5 ± 1.7 ^a^	14.0 ± 0.6 ^b^	13.1 ± 1.0 ^b^	<0.001
Delfinidin-3-*O*-glucoside	76.6 ± 2.9 ^b^	37.1 ± 0.6 ^c^	115.9 ± 4.0 ^a^	<0.001
Cyanidin-3-*O*-glucoside	35.7 ± 5.8 ^b^	13.1 ± 0.1 ^c^	59.3 ± 6.6 ^a^	<0.001
Malvidin-3-*O*-glucoside	35.7 ± 4.1 ^a^	16.2 ± 1.2 ^b^	13.5 ± 1.6 ^b^	<0.001
Peonidina-3-*O*-glucoside	2.6 ± 0.1 ^a^	0.0 ± 0.0 ^c^	1.5 ± 0.2 ^b^	<0.001
Petunidin-3-*O*-glucoside	45.3 ± 1.2 ^a^	28.4 ± 1.2 ^b^	25.5 ± 2.3 ^b^	<0.001
*Flavonols and stilbene*				
Kaempferol-3-*O*-glucoside	13.1 ± 0.1 ^a^	7.9 ± 0.1 ^b^	4.1 ± 0.2 ^c^	<0.001
Rutin	18.3 ± 0.1 ^a^	13.1 ± 0.1 ^b^	5.6 ± 0.5 ^c^	<0.001
Isorhamnetin-3-*O*-glucoside	15.7 ± 1.7 ^a^	10.0 ± 0.6 ^b^	7.6 ± 0.8 ^b^	<0.001
Myricetin	0.0 ± 0.0 ^c^	11.8 ± 0.1^a^	4.1 ± 0.2 ^b^	<0.001
*Trans*-resveratrol	7.8 ± 0.1 ^a^	5.2 ± 0.1 ^b^	1.3 ± 0.1 ^c^	<0.001
Quercetin-3-β-d-glucoside	99.3 ± 7.0 ^b^	138.4 ± 3.2 ^a^	17.2 ± 2.1 ^c^	<0.001
*Phenolic acid*				
Gallic acid	30.5 ± 2.3 ^a^	22.7 ± 1.5 ^b^	15.3 ± 2.7 ^c^	<0.001
Caftaric acid	32.2 ± 1.2 ^b^	86.0 ± 0.6 ^a^	24.6 ± 2.6 ^c^	<0.001
Caffeic acid	1.6 ± 0.1 ^b^	2.4 ± 0.1 ^a^	1.6 ± 0.2 ^b^	<0.001
Ferulic acid	18.3 ± 0.1 ^a^	10.5 ± 0.1 ^b^	5.9 ± 0.2 ^c^	<0.001
Chlorogenic acid	20.1 ± 1.2 ^a^	20.0 ± 0.6 ^a^	7.8 ± 0.8 ^b^	<0.001
*p*-Coumaric acid	33.1 ± 4.6 ^a^	30.6 ± 1.5 ^a^	15.5 ± 1.4 ^b^	<0.001
*Flavanols*				
(–)-Epicatechin	17.4 ± 1.2 ^b^	22.7 ± 1.5 ^a^	4.3 ± 0.4 ^c^	<0.001
(–)-Epicatechin gallate	68.8 ± 4.6 ^a^	48.5 ± 1.7 ^b^	35.8 ± 3.1 ^c^	<0.001
(–)-Epigalatocatechin gallate	68.8 ± 6.4 ^a^	44.5 ± 0.9 ^b^	22.7 ± 0.5 ^c^	<0.001
(+)-Catechin	30.5 ± 2.3 ^a^	26.6 ± 0.6 ^ab^	24.6 ± 2.7 ^b^	0.034
*Procyanidins*				
Procyanidin A2	20.9 ± 0.1 ^a^	15.7 ± 0.1 ^b^	5.9 ± 0.2 ^c^	<0.001
Procyanidin B1	36.6 ± 5.2 ^b^	62.9 ± 2.6 ^a^	13.8 ± 2.0 ^c^	<0.001
Procyanidin B2	20.0 ± 2.3 ^b^	77.7 ± 4.9 ^a^	20.0 ± 2.6 ^b^	<0.001
Total compounds	1337.2 ± 71.1 ^a^	1031.0 ± 29.2 ^b^	1351.5 ± 49.2 ^a^	<0.001

^‡^ The values of mean ± standard deviation (SD) correspond to averages from three replicates. Different letters on the same line indicate significant differences between the values (*p* < 0.05).

**Table 4:** Effect of the drying method using conventional oven-drying and freeze-dryingprocesses in the total phenolic, total flavonoid, total anthocyanin, and antioxidant activity of the BRS magna grape skin residue.

Parameters (% Dry Weight)	Method Applied ^‡^			
	Fresh Peel	Oven-Drying	Freeze-Drying	*p*-Value
Total phenolic (mg.GAE/100 g)	489.5 ± 1.8 ^a^	149.9 ± 1.4 ^b^	148.3 ± 0.9 ^b^	<0.001
Total anthocyanin (mg.CE/100 g)	124.9 ± 2.7 ^c^	150.7 ± 0.9 ^b^	260.1 ± 4.6 ^a^	<0.001
Total flavonoids (mg.QE/100 g)	2.6 ± 0.1 ^a^	1.8 ± 0.1 ^b^	1.3 ± 0.1 ^c^	<0.001
DPPH (EC_50_ in μg/mL)	1.3 ± 0.2 ^c^	3.8 ± 0.1 ^b^	5.0 ± 0.1 ^a^	<0.001
FRAP (μmol.Fe^2+^/g)	184.1 ± 8.9 ^a^	163.5 ± 10.9 ^a^	82.9 ± 9.3 ^b^	<0.001

^‡^ The values of mean ± standard deviation (SD) correspond to averages from three samples. Different letters on the same line indicate significant differences between the values (*p* < 0.05). DPPH, free radical-scavenging activity; FRAP, ferric reducing antioxidant power.

## Supplementary Materials

The Supplementary Materials are available online. Figure S1, Chromatograms of the phenolic compounds and their respective retention time (RT), equation and regression coefficient (R2). To identify and quantify the compounds were used DAD in the wavelengths 280 (a), 320 (b), 360 (c), 520 nm (d), and fluorescence at 280 nm excitation and 320 nm emission (e).
