# Supplementary material for: Effect of Drying Methods on Bioactive Compounds and Antioxidant Capacity in Grape Skin Residues from the New Hybrid Variety “BRS Magna”
Source: Molecules. 2020 Aug 14;25(16):3701. doi: 10.3390/molecules25163701 (PMC7466153; doi:10.3390/molecules25163701)
Supplement: Supplementary file 1 [file molecules-25-03701-s001.pdf]

molecules

Supplementary Materials

Effect of Drying Methods on Bioactive Compounds and Antioxidant Capacity in Grape Skin Residues from the New Hybrid Variety "BRS Magna"

Gabriela Viana da Silva [1], Bruna Aparecida Souza Machado [2], Walkia Polliana de Oliveira [1], Camilla Fernanda Godinho da Silva [1], Cedenir Pereira de Quadros [3], Janice Izabel Druzian [1], Ederlan de Souza Ferreira [1,*] and Marcelo Andrés Umsza-Guez [4,*]

[1] School of Pharmacy, Federal University of Bahia (UFBA), Salvador 40170-115, Bahia, Brazil; gvstecnologa@yahoo.com (G.V.d.S.); walkia2010@hotmail.com (W.P.d.O.); camillagodinho@gmail.com (C.F.G.d.S.); janicedruzian@hotmail.com (J.I.D.)

[2] Technology College, National Service for Industrial Learning (SENAI/CIMATEC), Salvador 41650-010, Bahia, Brazil; brunam@fieb.org.br

[3] School of Pharmacy, Federal University of the São Francisco Valley (UNIVASF), Petrolina 56300-000, Pernambuco, Brazil; cedenir.quadros@univasf.edu.br

[4] Department of Biotechnology, Health Science Institute, Federal University of Bahia (UFBA), Salvador 40170-115, Bahia, Brazil; marcelo.umsza@ufba.br (M.A.U.-G.)

* Correspondence: ederlan.ferreira@ufba.br (E.d.S.F.); marcelo.umsza@ufba.br (M.A.U.-G.); Tel.: +55-71-99231-3184(E.d.S.F.); Tel.: +55-71-99285-9330 (M.A.U-G.)

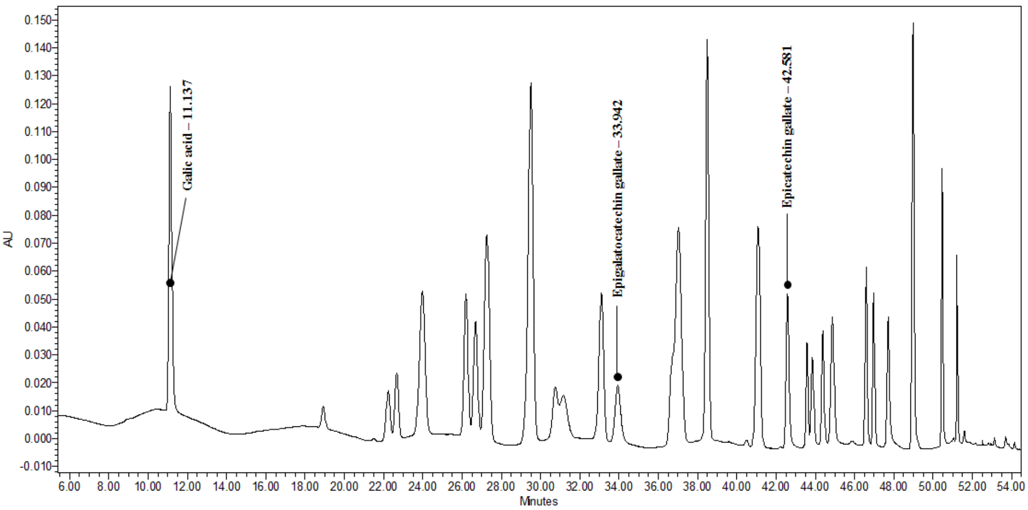

Compound	Retention time (min)	Equation	R^2
Gallic acid	11.137	y = 173091x + 10779	0.9987
(−)-Epicatechin gallate	42.581	y = 122591x − 2556.5	0.9962
(−)-Epigalatocatechin gallate	33.942	y = 132703x − 5981.3	0.9993

(**a**)

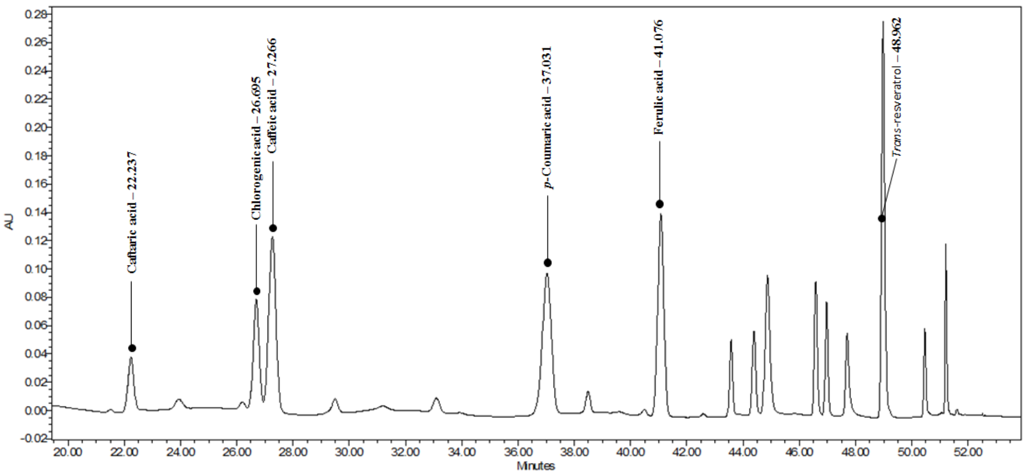

Compound	Retention time (min)	Equation	R^2
Trans-resveratrol	48.962	y = 147196x + 12633	0.9991
Caffeic acid	27.266	y = 121831x + 26031	0.9988
Caftaric acid	22.237	y = 113729x + 15360	0.9993
Ferulic acid	41.076	y = 58132x + 9098	0.9978
Chlorogenic acid	26.695	y = 65252x + 27046	0.9986
p-Coumaric acid	37.031	y = 137748x + 25549	0.9999

(**b**)

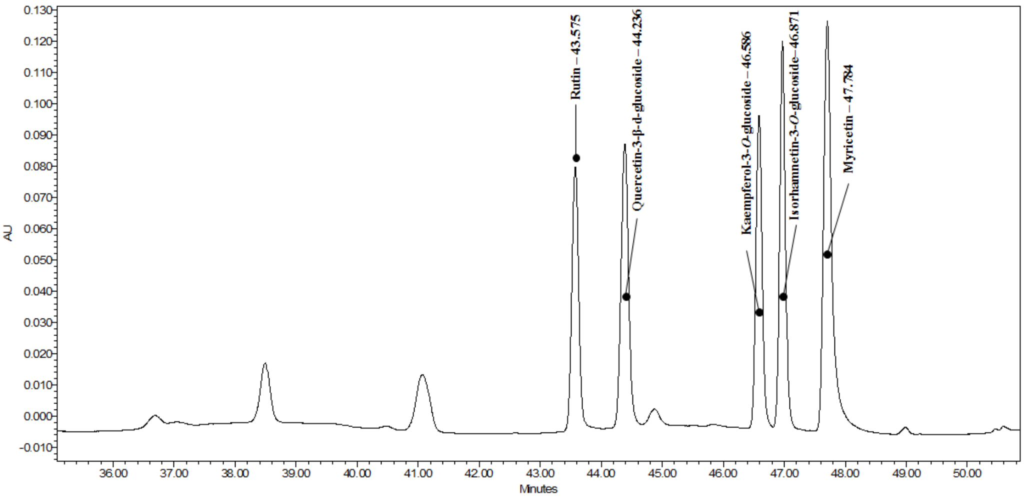

Compound	Retention time (min)	Equation	R^2
Kaempferol-3-*O*-glucoside	46.582	y = 25426x + 1397.2	0.9987
Myricetin	47.784	y = 75965x + 1050.5	0.9992
Isorhamnetin-3-*O*-glucoside	46.871	y = 43690x + 2006.7	0.9988
Rutin	43.575	y = 40716x + 1009.7	0.9998
Quercetin-3-β-d-glucoside	44.236	y = 83734x + 2500.1	0.9996

(**c**)

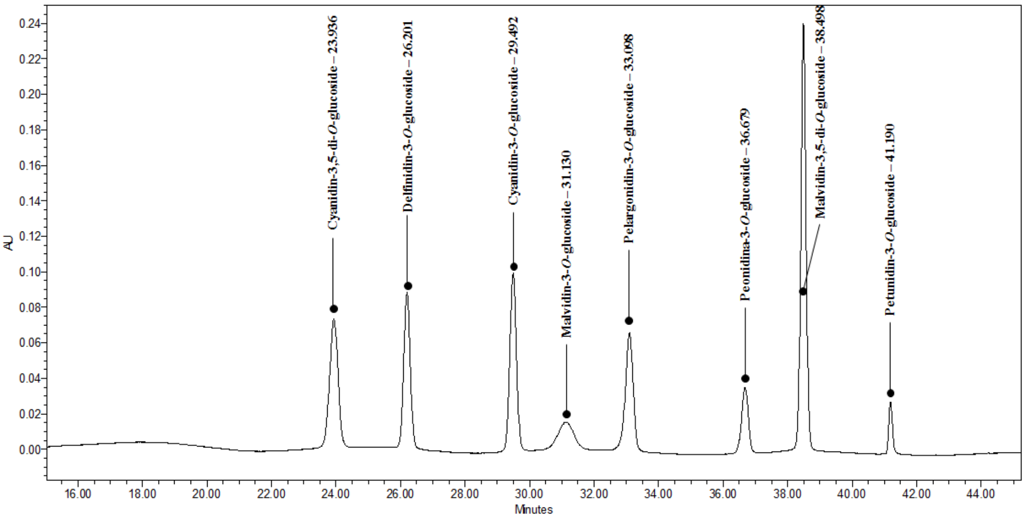

Compound	Retention time (min)	Equation	R^2
Cyanidin-3,5-di-*O*-glucoside	23.936	y = 22844x + 10748	0.9984
Malvidin-3,5-di-*O*-glucoside	38.498	y = 42295x + 16847	0.9990
Pelargonidin-3-*O*-glucoside	33.098	y = 55444x + 5843.9	0.9995
Delfinidin-3-*O*-glucoside	26.201	y = 37747x + 17541	0.9990
Cyanidin-3-*O*-glucoside	29.492	y = 45405x + 16107	0.9984
Malvidin-3-*O*-glucoside	31.130	y = 55142x + 9098	0.9976
Peonidina-3-*O*-glucoside	36.679	y = 67001x + 2076.4	0.9976
Petunidin-3-*O*-glucoside	41.190	y = 55444x + 5843.9	0.9995

(**d**)

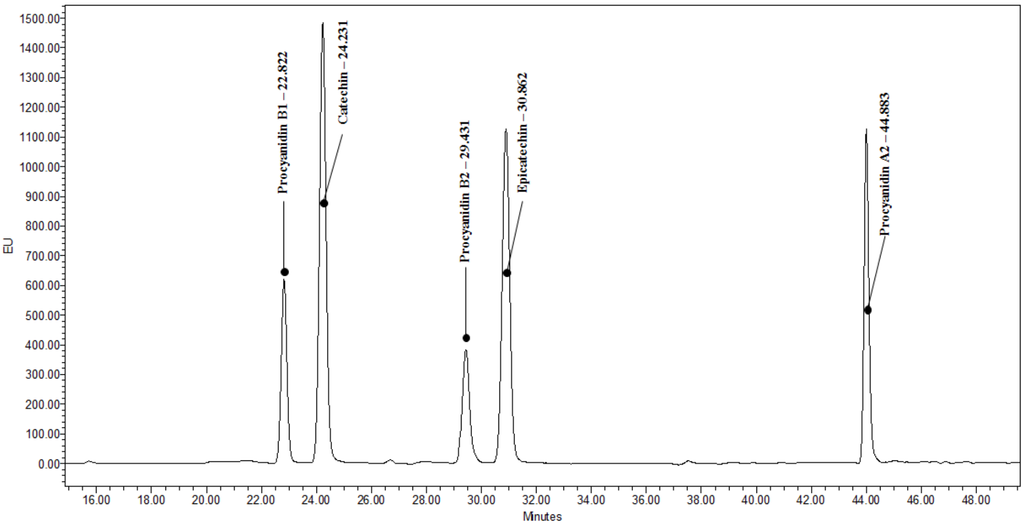

Compound	Retention time (min)	Equation	R^2
(–)-Epicatechin	30.862	y = 122591x − 2556.5	0.9962
(+)-Catechin	24.231	y = 1 × 107x + 6 × 106	0.9887
Procyanidin A2	44.883	y = 8 × 106x + 2 × 106	0.9901
Procyanidin B1	22.822	y = 8 × 106x + 2 × 106	0.9901
Procyanidin B2	29.431	y = 7 × 106x + 4 × 106	0.9839

(**e**)

Figure S1: Chromatograms of the phenolic compounds and their respective retention time (RT), equation and regression coefficient (R^2). To identify and quantify the compounds were used DAD in the wavelengths 280 (**a**), 320 (**b**), 360 (**c**), 520 nm (**d**), and fluorescence at 280 nm excitation and 320 nm emission (**e**).
